# A Study on the Neurological Mechanisms of the Impact of Different Dialysis Methods on Brain Structure and Functional Network Dynamic Reorganization in Patients With End‐Stage Renal Disease Undergoing Dialysis

**DOI:** 10.1002/brb3.70623

**Published:** 2025-06-18

**Authors:** Hui Han, Huijuan Chen, Ying Zhang, Jiali Wei

**Affiliations:** ^1^ Department of Nephrology Hainan General Hospital (Hainan Affiliated Hospital of Hainan Medical College) Haikou China

**Keywords:** brain structure, cognitive function, dialysis method, end‐stage renal disease, hemodialysis, peritoneal dialysis

## Abstract

**Introduction:**

End‐stage renal disease (ESRD) is a severe condition that necessitates dialysis. Dialysis treatments, including hemodialysis and peritoneal dialysis, can affect various organs, including the brain, leading to potential cognitive decline. This study investigates the neurological effects and brain network reorganization in ESRD patients undergoing different dialysis methods.

**Methods:**

A total of 120 ESRD patients were randomly divided into two groups: 60 patients undergoing hemodialysis and 60 undergoing peritoneal dialysis. Patients were followed for 1 year. Neuropsychological scales assessed cognitive function, and MRI scans were used to analyze brain structure and functional network dynamics before and after dialysis. Laboratory indicators and clinical outcomes were also recorded.

**Results:**

Both dialysis methods improved renal function, but peritoneal dialysis showed more favorable effects on cognitive function, with higher MMSE and MOCA scores. MRI analysis revealed better functional brain network efficiency in the peritoneal dialysis group. No significant differences were observed in the incidence of complications between the two groups.

**Conclusion:**

Although hemodialysis showed better renal function outcomes, peritoneal dialysis was associated with better cognitive performance and brain network reorganization. Both methods were safe, but peritoneal dialysis may offer neuroprotective benefits over hemodialysis in ESRD patients.

**Trial Registration:**

National Health Insurance Medical Research Registration and Filing Information System: MR‐46‐22‐012452

## Graphical abstract

Hemodialysis outperforms peritoneal dialysis in renal clearance and enhances brain default mode network activity, suggesting dual benefits of renal function improvement and neuroprotection.

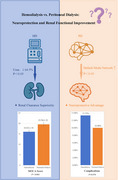

## 1 Introduction

End‐stage renal disease (ESRD) is the final stage of chronic kidney diseases, characterized by an annual incidence of approximately 30.7/100,000 and a mortality rate of about 20.9/100,000 in Chinese adults (Kanda et al. 2017). Dialysis, a commonly used renal replacement therapy for ESRD patients, is associated with various complications, including gas embolism, anticoagulant drug use, and disturbances in fluid, electrolyte, and acid–base balance. These complications can contribute to nerve damage and cognitive impairment in ESRD patients (Kanda et al. 2017; Moiseev et al. 2017). Surveys suggest that between 16%–38% of ESRD patients experience cognitive dysfunction (Tangren et al. 2018), with mechanisms linked to renal anemia, inadequate uremic toxin clearance, oxidative stress, inflammation, endothelial dysfunction, and hyperparathyroidism related to dialysis‐related complications (Wark 2020). While previous studies have examined the effects of dialysis on cerebral function and cognition (Choi et al. 2025; Chang et al. 2021), limited research has specifically compared the neurological outcomes of different dialysis methods. This study aims to fill that gap by exploring the neurobiological mechanisms underlying the impact of hemodialysis and peritoneal dialysis on brain structure and the dynamic reorganization of functional networks in ESRD patients. By systematically evaluating the comprehensive effects of these dialysis modalities, this research offers a more holistic understanding of how different dialysis methods influence neurological function in ESRD patients.

## 2 Materials and Methods

### 2.1 General Information

A total of 120 ESRD patients treated at our hospital from January 2021 to December 2023 were randomly divided into two groups using the envelope method: the hemodialysis group (*n* = 60) and the peritoneal dialysis group (*n* = 60). There were no statistically significant differences in general information between the two groups (*p* > 0.05), as shown in Table 1. Notably, both groups had a significantly higher number of female patients compared to males, with females comprising 75% (45/60) of the hemodialysis group and 70% (42/60) of the peritoneal dialysis group. This gender distribution aligns with the established demographic trend in ESRD populations, where women are more likely to develop kidney disease and require dialysis, especially among those with comorbidities such as diabetes or hypertension. Despite this, statistical analysis found no significant interactions between gender and the outcomes of interest, including cognitive function and brain network reorganization. Consequently, the higher proportion of female patients in both groups did not confound the results or affect the interpretation of the findings. In addition, potential confounding factors such as dialysis adequacy, patient compliance with treatment regimens, and other related clinical variables were closely monitored and adjusted for in the statistical analysis to ensure that any observed effects were not influenced by these factors. The study was approved by the ethics committee of Hainan General Hospital (Hainan Affiliated Hospital of Hainan Medical College) (No. 202092). This study has been registered in the National Health Insurance Medical Research Registration and Filing Information System under registration number MR‐46‐22‐012452. For further details, please refer to the following website: https://www.medicalresearch.org.cn/login.

**TABLE 1:** Comparison of general information.

Group	Gender		Age (years)	Education (years)	Duration of dialysis (months)	BMI (kg/m^2^)
	Male	Female				
Hemodialysis group (*n* = 60)	15 (25.00)	45 (75.00)	42.63 ± 16.89	11.79 ± 3.46	35.46 ± 12.02	22.16 ± 2.13
Peritoneal dialysis group (*n* = 60)	18 (30.00)	42 (70.00)	42.23 ± 15.10	11.98 ± 4.12	36.46 ± 11.08	22.13 ± 1.98
*χ* ^2^/*t*	0.376		0.137	−0.274	−0.474	0.080
*p*	0.540		0.891	0.785	0.636	0.936

Inclusion criteria: (1) patients aged 18–60 years. (2) Patients are right‐handed without visual or speech communication impairments. (3) No history of brain lesions or brain surgery. (4) Patients provide informed consent for this study.

Exclusion criteria: (1) patients with systemic diseases. (2) Patients with systemic inflammatory reaction diseases during treatment. (3) Patients with kidney damage due to hypertension, diabetes, or lupus nephritis. (4) Patients with hyperparathyroidism.

### 2.2 Methods

Hemodialysis group: Hemodialysis treatment is performed using autogenous arteriovenous fistula for blood dialysis. The dialysate solution consists of sodium chloride, potassium chloride, calcium chloride, magnesium chloride, and acetic acid solution. Blood flow rate is 200–250 mL/min, dialysate flow rate is 500 mL/min, duration is 4 h per session, and three sessions per week.

Peritoneal dialysis group: Peritoneal dialysis treatment is performed by inserting a straight tube through a paracentesis beside the navel, leaving a dialysis tube in the abdominal cavity. Baxter International Inc.’s peritoneal dialysis device and dialysate are used. The exchange volume is 2000 mL per session, three sessions per day.

Both groups are followed up for at least 12 months.

### 2.3 Observation Indicators

Record the renal function indicators of the two groups of patients before and after the final follow‐up: collect fasting venous blood samples from both groups of patients before and after the final follow‐up. Use a Hitachi 7600 Biochemical Analyzer and original reagents to test urea and blood creatinine. Calculate creatinine clearance rate as follows: creatinine clearance rate = (body weight × [140 − age])/(*a* × blood creatinine), where body weight is in kg, age is in years, blood creatinine is in mg/dL, and “a” is 72 for males and 85 for females.

Evaluate the overall cognitive function of patients using neurological psychology scales. Use the Mini‐Mental State Examination (MMSE) and the Montreal Cognitive Assessment (MOCA) to assess overall cognitive function. The MMSE is a widely used tool that evaluates memory, orientation, language ability, calculation ability, and attention, and is considered effective in screening for cognitive impairment in clinical settings (Folstein et al. 1975). The MOCA is particularly sensitive to mild cognitive impairment and assesses a broader range of cognitive domains including executive function, attention, language, memory, visuospatial skills, and orientation (Nasreddine et al. 2005). Both tools have been validated for use in patients with chronic illnesses, including ESRD, and are suitable for comparing cognitive outcomes in different dialysis modalities (Tiffin‐Richards et al. 2014; Kalirao et al. 2011). Use the Self‐Rating Anxiety Scale (SAS) and Self‐Rating Depression Scale (SDS) to assess anxiety and depression states (Roumeliotis et al. 2021).

MOCA assesses the patient's orientation, naming ability, executive ability, calculation ability, attention, abstract thinking ability, language ability, and delayed memory. MMSE evaluates the patient's memory, orientation, language ability, calculation ability, and attention. Higher scores indicate better cognitive ability. SAS and SDS assess patient anxiety and depression status, with higher scores indicating more severe anxiety and depression.

MRI examinations were performed on all patients before treatment and at the final follow‐up, utilizing the Siemens Skyra 3.0T superconducting MR imaging system. The global topological properties of the default brain functional network parameters, including global efficiency, local efficiency, clustering coefficient, and characteristic path length, were calculated before and after treatment. To ensure the robustness and inter‐rater reliability of the analysis, multiple trained raters independently processed the MRI data using standardized protocols. Inter‐rater reliability was assessed using the intra‐class correlation coefficient (ICC), with a threshold of ICC > 0.75 considered acceptable for data inclusion.

Record the occurrence of complications in both groups of patients and calculate the overall incidence.

### 2.4 Adjustment for Dialysis Adequacy and Patient Compliance

In this study, we closely monitored dialysis adequacy for both hemodialysis and peritoneal dialysis groups. Dialysis adequacy was assessed through regular measurements of the *K*_t_/*V* (for hemodialysis) and solute clearance (for peritoneal dialysis). Patient compliance was evaluated based on self‐reports and treatment records, ensuring adherence to prescribed treatment schedules. We also recorded and adjusted for relevant clinical factors, such as the presence of comorbid conditions, medication adherence, and any complications arising during the study period.

### 2.5 Statistical Analysis

Statistic Package for Social Science (SPSS) 23.0 software (IBM, Armonk, NY, USA) was used for data integration and analysis. Count data are expressed as (*n*/%), and the Chi‐square test is used. Measurement data are expressed as (± *s*), and the *t*‐test is used. A significance level of *α* < 0.05 indicates statistical significance.

## 3 Results

### 3.1 Renal Function Comparison

Before treatment, there was no significant difference in various renal function indicators between the two groups (*p* > 0.05). At the final follow‐up, the levels of urea and blood creatinine in patients were lower than before treatment, with the hemodialysis group lower than the peritoneal dialysis group. Creatinine clearance rate increased compared to before treatment, with the hemodialysis group higher than the peritoneal dialysis group (*p* < 0.05). See Table 2.

**TABLE 2:** Comparison of renal function.

Group	Urea (mmol/L)		Blood creatinine (mol/L)		Creatinine clearance (mL/min)	
	Before treatment	Last follow‐up	Before treatment	Last follow‐up	Before treatment	Last follow‐up
Hemodialysis group (*n* = 60)	28.77 ± 5.98	10.23 ± 4.15^*^	844.15 ± 62.12	325.46 ± 31.54^*^	28.66 ± 7.15	42.15 ± 7.26^*^
Peritoneal Dialysis Group (n = 60)	28.45 ± 6.12	16.77 ± 5.12^*^	842.16 ± 54.89	402.22 ± 45.02^*^	28.67 ± 6.19	35.45 ± 7.15^*^
*t*	0.290	−7.686	0.186	−10.817	−0.008	5.093
*p*	0.773	< 0.001	0.853	< 0.001	0.993	< 0.001

*Note*: *Significant at *p* < 0.05 when compared with the same group before treatment.

### 3.2 MMSE and MOCA Scale Scores

Before and after treatment comparison: there was no difference in cognitive function scores between the two groups of patients before treatment (*p* > 0.05). After treatment, the MMSE and MOCA scores of the patients decreased, and the scores of the peritoneal dialysis group were higher than those of the hemodialysis group (*p* < 0.05). See Table 3.

**TABLE 3:** Comparison of MMSE and MOCA scores before and after treatment.

Group	MOCA score (points)		MMSE score (points)	
	Before treatment	Last follow‐up	Before treatment	Last follow‐up
Hemodialysis group (*n* = 60)	23.02 ± 2.04	16.01 ± 1.56^*^	22.98 ± 2.12	16.52 ± 1.08^*^
Peritoneal Dialysis Group (n = 60)	23.11 ± 1.85	19.56 ± 1.12^*^	22.78 ± 2.45	19.81 ± 1.23^*^
*t*	−0.253	−14.319	0.478	−15.569
*p*	0.801	< 0.001	0.633	< 0.001

*Note*: *Significant at *p* < 0.05 when compared with the same group before treatment.

### 3.3 Comparison of SAS and SDS Scores

Before and after treatment: there was no difference in anxiety and depression scores between the two groups of patients before and after treatment (*p* > 0.05). At the final follow‐up, both the hemodialysis and peritoneal dialysis groups exhibited increased SAS and SDS scores compared to before treatment (*p* < 0.05). Notably, the hemodialysis group showed higher posttreatment SAS and SDS scores than the peritoneal dialysis group, indicating a greater increase in anxiety and depression levels. See Table 4.

**TABLE 4:** Comparison of SAS and SDS scores before and after treatment.

Group	SAS score (points)		SDS score (points)	
	Before treatment	Last follow‐up	Before treatment	Last follow‐up
Hemodialysis group (*n* = 60)	34.12 ± 3.12	53.44 ± 6.15^*^	36.44 ± 3.16	54.12 ± 6.46^*^
Peritoneal dialysis group (*n* = 60)	33.89 ± 2.41	45.02 ± 5.12^*^	36.12 ± 5.42	47.26 ± 4.55^*^
*t*	0.452	1.375	0.395	0.843
*p*	0.652	0.172	0.693	0.401

*Note*: *Significant at *p* < 0.05 when compared with the same group before treatment.

### 3.4 Comparison of Global Topological Parameters of Brain Functional Default Network

Before and after treatment: before treatment, there was no difference in global efficiency, local efficiency, clustering coefficient, and characteristic path length between the two groups of patients (*p* > 0.05). At the final follow‐up, the global efficiency, local efficiency, and clustering coefficient decreased compared to before treatment, and the peritoneal dialysis group was higher than the hemodialysis group. The characteristic path length increased compared to before treatment, and the peritoneal dialysis group was lower than the hemodialysis group (*p* < 0.05). See Table 5.

**TABLE 5:** Comparison of global topological parameters of default mode network brain function before and after treatment.

Group	Global efficiency		Local efficiency	
	Before treatment	Last follow‐up	Before treatment	Last follow‐up
Hemodialysis group (*n* = 60)	0.18 ± 0.03	0.11 ± 0.04^*^	0.22 ± 0.02	0.14 ± 0.04^*^
Peritoneal dialysis group (*n* = 60)	0.17 ± 0.04	0.13 ± 0.05^*^	0.23 ± 0.04	0.17 ± 0.08^*^
*t*	1.549	−2.419	−1.732	−2.598
*p*	0.124	0.017	0.086	0.011
Group	Clustering coefficient		Characteristic path length	
	Before treatment	Last follow‐up	Before treatment	Last follow‐up
Hemodialysis group (*n* = 60)	0.28 ± 0.06	0.16 ± 0.08^*^	6.22 ± 0.38	8.99 ± 0.32^*^
Peritoneal dialysis group (*n* = 60)	0.29 ± 0.05	0.22 ± 0.06^*^	6.13 ± 0.56	8.78 ± 0.31^*^
*t*	−0.992	−4.648	1.030	3.651
*p*	0.323	< 0.001	0.305	< 0.001

*Note*: *Significant at *p* < 0.05 when compared with the same group before treatment.

### 3.5 Incidence of Complications

There was no difference in the incidence of complications between the two groups of patients (*p* > 0.05). See Table 6.

**TABLE 6:** Comparison of the occurrence of complications.

Group	Cacotrophy	Cardiovascular accident	Cerebrovascular accident	Infect	Total occurrence
Hemodialysis group (*n* = 60)	1 (1.67)	2 (3.33)	2 (3.33)	3 (5.00)	8 (13.33)
Peritoneal dialysis group (*n* = 60)	2 (3.33)	1 (1.67)	1 (1.67)	2 (3.33)	6 (10.00)
*χ* ^2^					0.323
*p*					0.570

## 4 Discussion

Currently, renal replacement therapies include hemodialysis, peritoneal dialysis, and kidney transplantation. Due to the limitations of kidney transplantation, such as high cost, shortage of kidney sources, and the possibility of postoperative rejection reactions, its clinical application is not as widespread as dialysis (O'Connor and Corcoran 2012). Both peritoneal dialysis and hemodialysis can effectively remove toxic metabolic products from the patient's body (Collins 2003). In this study, it was found that the levels of urea and blood creatinine decreased, and creatinine clearance increased after treatment, with the hemodialysis group showing lower levels and higher clearance rates. However, it is important to note that the follow‐up period of 1 year may not fully capture the long‐term cognitive or neurological changes in ESRD patients. Longer follow‐up studies are needed to determine whether the cognitive improvements observed with peritoneal dialysis are sustained over time and to assess any delayed effects on brain function. This result suggests that the treatment effect of hemodialysis is slightly better than that of peritoneal dialysis. Hemodialysis involves establishing extracorporeal circulation to filter excess electrolytes and toxic metabolic products from the blood before returning it to the body (Carlo et al. 2015). Peritoneal dialysis uses the semi‐permeable nature of the patient's own peritoneum to eliminate waste fluid via diffusion and convection, and its efficiency in removing metabolic products is slightly lower than that of hemodialysis (Carlo et al. 2015; Mojcik and Klippel 1996).

In clinical practice, it has been observed that although dialysis can prolong the survival time of ESRD patients, it may lead to a decrease in their quality of life. Patients often develop anxiety, depression, and cognitive impairment, significantly impacting their quality of life (Georgianos et al. 2018). Cognitive impairment in ESRD patients undergoing dialysis mainly manifests as a decline in cognitive abilities such as orientation, learning and memory, and executive function. It can affect treatment compliance and diminish self‐management capabilities, thereby influencing the treatment of kidney disease (Georgianos et al. 2018; Sawinski and Murphy 2008). While this study found that the MMSE and MOCA scores of patients decreased after treatment, with the peritoneal dialysis group showing higher scores than the hemodialysis group, it is important to note that these findings are specific to the patients enrolled in this study. The study sample was drawn from a single healthcare facility with a specific patient demographic, which may not be representative of the broader ESRD population. Moreover, the results may not be directly applicable to ESRD patients in diverse healthcare settings, where variables such as healthcare access, socioeconomic factors, and comorbid conditions may differ. Future research involving more diverse populations and healthcare settings is needed to confirm the generalizability of these findings. This may be due to several factors. Peritoneal dialysis may confer cognitive benefits because it avoids the systemic inflammatory responses associated with the biocompatibility of hemodialysis membranes, which can damage vascular endothelial integrity and exacerbate central nervous system injury. In addition, the maintenance of stable hemodynamics during peritoneal dialysis reduces the risk of cerebral hypoxia and ischemia, which can impair cognitive function. The reduced need for extracorporeal circulation in peritoneal dialysis may also decrease oxidative stress, a key factor in neurodegeneration, and improve neurovascular health by promoting better blood–brain barrier integrity. Furthermore, peritoneal dialysis has been shown to reduce amyloid deposition, a known contributor to cognitive decline, which may explain the improved cognitive outcomes in this patient population (Hallan and Vikse 2008; Imam and Coleman 2019). Peritoneal dialysis, which does not require extracorporeal circulation, reduces the impact on the body's internal environment and maintains stable hemodynamics during treatment, resulting in less influence on the blood supply and oxygenation of the central nervous system. Therefore, peritoneal dialysis causes less damage to cognitive function (Lavergne et al. 2021; Müller and Goldstein 2011).

This study also found that both groups of patients experienced an increase in anxiety and depression scores after treatment, with no significant difference between the two groups. This result suggests that both types of dialysis can induce anxiety and depression in patients, with similar effects on negative emotions. This is because the discomfort caused by kidney disease itself, concerns about prognosis, and the pain generated during treatment can all lead to negative emotions in ESRD patients (Agarwal 2016; Hood and Gennari 1996). Both peritoneal dialysis and hemodialysis can cause certain discomfort, and their impact on patient emotions is not significantly different. However, as mentioned in the Section 2, there may still be unmeasured confounding factors, such as education level and baseline cognitive function, that could potentially influence cognitive outcomes. Future studies should aim to control for these confounding factors, possibly through matching techniques or multivariate regression analyses, to further validate the impact of different dialysis methods on cognitive function.

MRI examinations can reflect local brain activity and functional connections. The use of multimodal magnetic resonance analysis techniques, such as structural connectivity, functional connectivity, and graph theory analysis, can reflect the structure, functional networks, and specific brain areas related to cognitive function (Halen et al. 2012; Van Buren 2016). Global efficiency and local efficiency reflect the proficiency of information transmission in brain networks at global and local levels, respectively. The clustering coefficient reflects the local connectivity of the brain network, and characteristic path length reflects the level of global efficiency of the network (Cheigh and Stenzel 1993; Halen et al. 2012). Detecting these indicators helps objectively reflect local brain activity and the degree of functional connectivity, thereby objectively reflecting the degree of cognitive impairment. This study found that after treatment, the global efficiency, local efficiency, and clustering coefficient of patients decreased, with the peritoneal dialysis group showing higher values than the hemodialysis group. In addition, the characteristic path length increased posttreatment, with the peritoneal dialysis group demonstrating lower values compared to the hemodialysis group (Hingorani and Watkins 2000). However, it is important to note that the absence of a non‐dialysis or healthy control group limits the interpretation of these findings. Without such a control group, it is challenging to determine whether the observed brain changes are specifically due to dialysis or represent part of a broader pattern of cognitive decline in ESRD patients. This result suggests that peritoneal dialysis has a positive impact on the dynamic reorganization of brain functional networks, improving local brain activity and functional connectivity, and is more conducive to neuroprotection. This may be because the biocompatibility of the hemodialysis membrane is inferior to that of peritoneal dialysis, and long term hemodialysis can induce chronic inflammatory reactions, affecting nutritional metabolism and increasing the risk of renal anemia. Chronic anemia can lead to cerebral ischemia and hypoxia, reduce the activity of brain cells' redox enzymes, and ultimately affect cognitive function.

This study also found that the incidence of complications did not differ between the two groups of patients, indicating that the safety of the two dialysis methods is comparable.

In conclusion, while the treatment effect of hemodialysis is slightly better than that of peritoneal dialysis, peritoneal dialysis demonstrates a positive impact on cognitive function and the dynamic reorganization of brain functional networks, making it more conducive to neuroprotection. Both dialysis methods exhibit a high level of safety. However, factors such as dialysis adequacy, patient compliance, and other clinical variables could have influenced these results. The study accounted for these factors by adjusting for dialysis adequacy (assessed via *K*_t_/*V* and solute clearance), patient compliance, and relevant comorbid conditions. Despite this, further studies, particularly those involving non‐dialysis or healthy control groups, are essential to provide a clearer understanding of how these treatments specifically impact brain function in ESRD patients compared to healthy individuals or those with other conditions. Such research could offer deeper insights into the relationship between dialysis methods and neurological outcomes in this patient population.

## 5 Limitation

The relatively short follow‐up period may not fully capture long term cognitive and neurological changes. Conducted at a single healthcare facility, the findings may not be entirely generalizable to broader ESRD populations. In addition, factors like patient compliance and pre‐existing cognitive function, which weren't fully controlled, might have influenced the results. These points suggest that future studies should involve larger, more diverse samples and account for baseline cognitive function differences.

## Author Contributions

**Hui Han**: conceptualization, formal analysis, funding acquisition, writing–original draft. **Huijuan Chen**: data curation, methodology, resources, writing–review and editing. **Ying Zhang**: data curation, formal analysis, writing–original draft. **Jiali Wei**: conceptualization, investigation, project administration, supervision, writing–review and editing.

## Ethics Statement

The study was approved by the ethics committee of Hainan General Hospital (Hainan Affiliated Hospital of Hainan Medical College) (No. 202092).

## Consent

Informed consent has been obtained from all of the participants.

## Conflicts of Interest

The authors declare no conflicts of interest.

## Peer Review

The peer review history for this article is available at https://publons.com/publon/10.1002/brb3.70623

## Data Availability

The data that support the findings of this study are available on request from the corresponding author. The data are not publicly available due to privacy or ethical restrictions.
